# Molecular and Neuroimaging Correlates of Bipolar Disorder: Linking Inflammation, Mitochondria, and Brain Circuitry

**DOI:** 10.3390/ijms27031478

**Published:** 2026-02-02

**Authors:** Ewa Alicja Ogłodek, Jan Vober, Martin Hýža

**Affiliations:** 1 Collegium Medicum, Jan Dlugosz University in Częstochowa, Waszyngtona 4/8 Street, 42-200 Częstochowa, Poland; 2 Department of Psychiatry, University Hospital Ostrava, 17. listopadu 1790, Poruba, 708 52 Ostrava, Czech Republic; 3 Department of Clinical Neurosciences, Faculty of Medicine, University of Ostrava, 708 52 Ostrava, Czech Republic

**Keywords:** biomarkers, bipolar disorder, cortico-limbic circuitry, inflammation, mitochondrial dysfunction, neuroimaging, precision medicine

## Abstract

Bipolar affective disorder (BD) is a severe mental illness characterized by recurrent episodes of mania, hypomania, and depression, accompanied by progressive neurobiological changes that go beyond the classical concepts of neurotransmitter dysregulation. Increasing evidence points to the key role of the interaction between inflammatory processes, mitochondrial dysfunction, and disturbances within neural networks in the pathogenesis, course, and treatment response of BD. Neuroinflammatory processes, including elevated levels of pro-inflammatory cytokines, chemokines, and microglial activation, are consistently reported in patients with BD and linked to cognitive impairment, accelerated neuroprogression, and treatment resistance. At the same time, mitochondrial abnormalities—such as impaired oxidative phosphorylation, excessive production of reactive oxygen species, and disturbances in calcium homeostasis—contribute to oxidative stress, synaptic dysfunction, and increased neuronal vulnerability, forming the biological substrate of mood instability. Findings from neuroimaging studies provide consistent evidence of structural and functional alterations within the cortico-limbic networks regulating emotions, including the prefrontal cortex, anterior cingulate cortex, amygdala, and hippocampus. Importantly, a growing number of studies demonstrate correlations between neuroimaging changes and inflammatory and metabolic biomarkers, making it possible to link molecular pathology with dysfunctions at the level of neural networks. The use of multimodal methods—encompassing structural and functional magnetic resonance imaging, spectroscopy, and molecular analyses—allows for a more precise explanation of these complex interactions and the identification of biomarkers of clinical states, progression, and treatment response. This review synthesizes current knowledge on the molecular and neuroimaging correlates of BD, emphasizing the interdependence of inflammatory processes, mitochondrial function, and neural networks. The integration of molecular biomarkers with imaging-based phenotyping opens new perspectives for precision medicine in BD.

## 1. Introduction

Bipolar disorder (BD) is a severe and highly heterogeneous psychiatric condition associated with recurrent episodes of mania or hypomania and depression, resulting in profound disturbances of mood, energy, and psychomotor activity. According to the Diagnostic and Statistical Manual of Mental Disorders, Fifth Edition (DSM-5), manic episodes are characterized by persistently elevated, expansive, or irritable mood accompanied by increased activity or energy, along with symptoms such as grandiosity, reduced need for sleep, pressured speech, flight of ideas, distractibility, psychomotor agitation, and engagement in high-risk behaviors. Depressive episodes, in contrast, are marked by anhedonia, sleep and appetite disturbances, fatigue, cognitive impairment, feelings of worthlessness, and, in severe cases, suicidal ideation. DSM-5 additionally recognizes mixed states, in which manic or hypomanic and depressive symptoms co-occur, conferring a particularly high risk of suicide and illness recurrence [1].

Beyond episodic mood instability, increasing evidence indicates that BD is a multisystem disorder involving dysregulation of immune, metabolic, and bioenergetic pathways. Two principal subtypes are distinguished: bipolar I disorder, defined by the presence of at least one full manic episode, and bipolar II disorder, characterized by hypomanic episodes and major depression without a history of mania [2]. Despite differences in symptom severity, both subtypes are associated with recurrent illness, cognitive deficits, and substantial psychosocial burden. Epidemiological studies estimate a lifetime prevalence of 1–2% for BD, rising to 4–5% when the broader bipolar spectrum is considered. The disorder typically emerges in late adolescence or early adulthood and often follows a progressive course marked by increasing episode frequency, treatment resistance, and persistent functional impairment [3].

BD is associated with a markedly elevated risk of suicide, with lifetime attempt rates of 25–50% and mortality rates of 10–15%, as well as increased prevalence of cardiometabolic and autoimmune comorbidities. Diagnostic delays of 5–10 years are common, largely due to the predominance of depressive symptoms and overlap with unipolar depression, resulting in inappropriate treatment and accelerated disease progression. These clinical observations further support the concept of BD as a systemic disorder rather than a purely affective illness [4,5].

At the molecular level, growing evidence implicates chronic low-grade inflammation, immune dysregulation, mitochondrial dysfunction, oxidative stress, and impaired synaptic plasticity as central mechanisms in BD pathophysiology [6,7,8]. Dysregulation of inflammasome signaling, particularly involving the NOD-like receptor protein 3 (NLRP3) complex, has been linked to neuroinflammatory processes affecting neuronal viability and mood regulation. In parallel, alterations in mitochondrial bioenergetics and redox homeostasis contribute to reduced neuronal resilience and disrupted neurotransmission [9,10,11,12].

Neuroimaging studies provide convergent evidence for these molecular abnormalities at the systems level. Structural and functional magnetic resonance imaging have revealed volume reductions and connectivity disturbances in the prefrontal cortex, hippocampus, and fronto-limbic circuits, as well as dysfunction within large-scale networks such as the default mode and salience networks. Positron emission tomography has enabled in vivo assessment of cerebral metabolism, neurotransmitter systems, and neuroinflammatory markers, thereby linking molecular pathology with functional brain alterations. Complementary methods, including transcranial Doppler ultrasonography, offer additional insights into cerebral hemodynamics across different phases of the disorder [13,14,15].

The integration of molecular and neuroimaging data represents a major challenge and opportunity in BD research. Advances in multi-omics approaches—encompassing genomics, epigenomics, transcriptomics, proteomics, and metabolomics—enable systematic mapping of molecular alterations and their association with neural circuits and clinical phenotypes. Such integrative strategies are essential for identifying robust biomarkers and advancing precision medicine in psychiatry [16,17,18].

Accordingly, this review aims to synthesize current evidence on the molecular mechanisms underlying bipolar disorder and their neuroimaging correlates. Specifically, we focus on immune and inflammatory pathways, mitochondrial dysfunction and oxidative stress, genetic and epigenetic regulation, and neuroimaging markers reflecting these processes at the network level. By integrating molecular and systems-level findings, this review seeks to contribute to the development of coherent pathophysiological models and to support biomarker discovery and personalized therapeutic strategies in bipolar disorder [19].

In summary, this review addresses current challenges in the molecular and neuroimaging characterization of bipolar disorder and outlines future directions toward biomarker-driven and precision psychiatry approaches.

## 2. Inflammation and Immune Dysregulation in Bipolar Disorder

Over the past two decades, research on BD has increasingly emphasized the role of inflammatory processes and immune dysregulation in its pathophysiology. From the perspective of contemporary psychiatry, BD is not merely an affective disorder characterized by cyclical mood changes, but rather a complex neuroimmunological disease leading to cognitive decline, structural brain changes, and progressive treatment resistance [20]. Central to this concept are cytokines and chemokines—immune mediators that facilitate communication between the immune system and the central nervous system. An imbalance between pro- and anti-inflammatory cytokines, excessive activation of the NLRP3 inflammasome, microglial reactivity, and astrocytic dysfunction together form the characteristic picture of chronic neuroinflammation. These processes impair neuroplasticity, destabilize neurotransmission, and disrupt neural networks. Meta-analyses and cohort studies confirm that patients with BD consistently show elevated levels of pro-inflammatory cytokines—IL-1β, IL-6, TNF-α, Interferon Gamma (IFN-γ), and Interleukin-17 (IL-17)—both during acute phases (mania, depression) and in periods of remission [21,22]. Interleukin-6 plays a particularly important role, as its elevation correlates with symptom severity, more frequent relapses, and poorer response to mood stabilizers such as lithium and valproate. Similar associations have been demonstrated for IL-1β, whose inflammasome-mediated activation contributes to depressive symptoms, reduced neuroplasticity, and increased suicide risk. Interleukin-18, in turn, drives chronic microglial activation and enhanced inflammatory responses, potentially leading to neurodegeneration and disease progression [23]. TNF-α further affects blood–brain barrier integrity and glutamatergic transmission, reinforcing excitotoxic mechanisms and increasing neuronal susceptibility to oxidative stress and mitochondrial damage. Anti-inflammatory cytokines are also crucial. Studies have shown reduced levels of Interleukin-10 (IL-10) and TGF-β, which under normal conditions suppress excessive immune responses and limit neurotoxicity [24]. Their deficiency promotes chronic inflammation and persistent immune disturbances.

Molecular biomarkers thus provide a mechanistic link between immune dysregulation and the neuroprogressive changes observed in bipolar disorder. Chronic activation of inflammatory pathways is associated not only with elevated cytokine levels but also with sustained microglial activation, which may persist beyond acute mood episodes. This prolonged immune activation is thought to underlie long-term alterations in synaptic remodeling, impaired neuroplasticity, and progressive structural brain changes within fronto-limbic circuits implicated in affective regulation. Consequently, inflammatory and immune-related biomarkers are increasingly considered valuable tools for monitoring disease progression, identifying biologically distinct subtypes of bipolar disorder, and predicting treatment response [25,26,27].

Chemokines also play an important role in regulating leukocyte migration—patients with BD display elevated levels of Monocyte Chemoattractant Protein-1 (MCP-1), C-C Motif Chemokine Ligand 2 (CCL2), Regulated on Activation, Normal T-cell Expressed and Secreted (RANTES), C-C Motif Chemokine Ligand 5 (CCL5), C-X-C Motif Chemokine Ligand 8 (CXCL8), Interleukin-8 (IL-8; CXCL8) [25]. MCP-1 facilitates monocyte infiltration into the CNS, thereby enhancing microglial activation, while fractalkine (CX3CL1), which mediates neuron–microglia communication, shows abnormal expression patterns in BD [28]. Collectively, these findings point to activation of the cytokine–chemokine axis. Excessive cytokine production in BD originates from activation of the NLRP3 inflammasome—an intracellular protein complex that detects Damage-Associated Molecular Patterns (DAMPs) and oxidative stress. Its activation triggers caspase-1 and the release of active forms of IL-1β and IL-18, thereby amplifying inflammation [28]. Elevated IL-18 levels have been confirmed in BD patients, correlating with episode frequency and disease severity [29]. Overactivation of NLRP3 is closely linked to mitochondrial dysfunction and the overproduction of reactive oxygen species (ROS), both hallmark features of BD [30].

Microglia represent the primary effectors of chronic immune activation. Positron Emission Tomography studies using translocator protein 18 kDa (TSPO) ligands have demonstrated increased microglial activity in the prefrontal cortex, anterior cingulate cortex, and hippocampus, particularly during manic phases. Activated microglia release cytokines, nitric oxide, and glutamate, which drive neurotoxicity and synaptic dysfunction. Animal models further confirm that chronic stress—a major risk factor for BD—induces microglial proliferation and NLRP3 activation, linking immune processes with behavioral alterations. Astrocytes, key regulators of brain homeostasis, have also received growing attention. They mediate glutamate uptake, regulate cerebral blood flow, and maintain blood–brain barrier integrity. Postmortem studies of BD patients have revealed reduced expression of astrocytic markers such as Glial Fibrillary Acidic Protein (GFAP) and glutamate transporters—Excitatory Amino Acid Transporter 1/2 (EAAT1/2), indicating impaired regulation of glutamatergic balance [31]. Astrocytes also produce inflammatory mediators—in BD, increased secretion of IL-6, MCP-1, and S100 calcium-binding protein B (S100B) has been observed, the latter being a marker of glial damage and activation, with levels correlating with episode severity and cognitive deficits [32]. Astrocytic dysfunction thereby promotes excitotoxicity and reorganization of neural circuits within fronto-limbic networks.

Beyond immune-mediated mechanisms, increasing evidence indicates that disturbances in cellular energy metabolism represent a critical and complementary dimension of bipolar disorder pathophysiology, directly linking molecular dysfunction with neuroprogressive and clinical outcomes [33,34].

Another key area involves bioenergetic disturbances and mitochondrial dysfunction. Neurons are exceptionally sensitive to energy deficits, with their proper functioning depending on efficient respiratory chain activity and Adenosine Triphosphate (ATP) production. In BD, reduced activity of mitochondrial enzymes, excessive production of reactive oxygen species (ROS), and elevated levels of oxidative stress markers such as malondialdehyde (MDA) have been observed [35]. These disturbances may lead to Deoxyribonucleic Acid (DNA) damage, membrane dysfunction, and impaired neuroplasticity, reflected clinically in cognitive deficits, emotional lability, and increased susceptibility to relapse [36,37].

Chronic immune activation in BD exerts profound effects on neuroplasticity and modifies the clinical picture. Pro-inflammatory cytokines reduce the expression of neurotrophic factors, particularly Brain-Derived Neurotrophic Factor (BDNF), whose levels are decreased in both depressive and manic phases [38]. BDNF deficiency contributes to cognitive deficits and emotional disturbances. Persistent inflammation also disrupts neurotransmitter metabolism—activation of the enzyme Indoleamine 2,3-Dioxygenase (IDO) shifts tryptophan metabolism toward the kynurenine pathway, leading to the production of neurotoxic metabolites such as quinolinic acid, an *N*-methyl-D-aspartate (NMDA) receptor agonist that induces depressive symptoms, anhedonia, and cognitive decline [39]. In parallel, TNF-α and IL-1β enhance glutamatergic transmission and weaken GABAergic activity, destabilizing neural networks and contributing to mood instability. Clinical studies show that patients with a heightened inflammatory profile are more likely to exhibit rapid cycling, respond poorly to mood stabilizers, and carry a greater risk of suicide [40]. Neuroimaging findings indicate that inflammatory markers correlate with hippocampal atrophy, cortical thinning, and white matter damage. Functional MRI studies reveal disrupted connectivity within the default mode network and salience network, linking chronic inflammation to dysfunctions in emotional regulation circuits.

The recognition of inflammation as a core mechanism in BD has opened new therapeutic avenues. Anti-inflammatory agents such as minocycline, celecoxib, and omega-3 fatty acids show beneficial effects as adjunctive treatments, reducing depressive symptoms and improving response to mood stabilizers. Lithium—the classic mood stabilizer—inhibits NLRP3 activation via Glycogen Synthase Kinase-3 beta (GSK-3β) blockade, suggesting that part of its clinical efficacy may derive from anti-inflammatory properties [41]. Increasing attention is also being paid to lifestyle interventions, such as physical activity and anti-inflammatory diets, which can reduce C-reactive protein (CRP) and pro-inflammatory cytokine levels.

In summary, inflammation and immune dysregulation represent central pathophysiological mechanisms in BD. The imbalance between pro- and anti-inflammatory cytokines, excessive NLRP3 activation, microglial reactivity, and astrocytic dysfunction together define a state of chronic inflammation that disrupts neuroplasticity, neurotransmission, and brain network function. These mechanisms explain the clinical manifestations of BD—mood instability, cognitive deficits, and high suicide risk—as well as its neuroprogressive nature. The integration of molecular, immunological, and neuroimaging data enables the development of new disease models and paves the way for personalized psychiatry, where inflammatory and mitochondrial biomarkers may serve as diagnostic, prognostic, and therapeutic indicators.

## 3. Mitochondrial Dysfunction and Oxidative Stress

In recent years, it has been firmly established that mitochondrial dysfunction and oxidative stress are key components of the pathophysiology of BD. Moving away from a reductionist model based solely on neurotransmission has enabled the reconceptualization of BD as a multisystem disorder, in which bioenergetic deficits, oxidative damage, and neurodegenerative changes coexist [42]. Mitochondria—responsible for ATP production via oxidative phosphorylation, regulation of calcium homeostasis, control of apoptosis, and generation of reactive oxygen species (ROS)—lie at the center of these processes. Their dysfunction results in energy deficiency and accumulation of toxic byproducts, which damage lipids, proteins, and DNA, thereby promoting neuroprogression and cognitive decline in patients with BD [43]. Postmortem evidence and molecular analyses have demonstrated reduced activity of complexes I and IV of the respiratory chain, decreased mitochondrial DNA (mtDNA) copy number, and mutations in genes encoding mitochondrial proteins [44,45].

These findings are consistent with MRS observations showing reduced phosphocreatine and ATP levels in the prefrontal cortex and caudate nucleus. Bioenergetic deficits disrupt neuronal excitability, synaptic transmission, and signal integration within neural networks, contributing to cognitive and emotional symptoms. A critical element of the pathomechanism is dysregulation of calcium homeostasis. In BD, abnormal mitochondrial Ca^2+^ uptake has been described, which decouples the activity–energy demand relationship [46]. Excessive Ca^2+^ accumulation triggers opening of the mitochondrial permeability transition pore (mPTP), mitochondrial membrane depolarization, and cytochrome c release, activating the apoptotic cascade. This leads to neuronal loss and degeneration of structures essential for mood regulation (hippocampus, prefrontal cortex). Bioenergetic disturbances are closely tied to increased oxidative stress. In BD, both overproduction of ROS (superoxide anion, H_2_O_2_, hydroxyl radical) and reduced activity of antioxidant systems (SOD, catalase, glutathione peroxidase) are observed. Consequences include lipid peroxidation (increased Malondialdehyde-MDA, 4-Hydroxynonenal-4-HNE), oxidative protein modifications (protein carbonyls, nitrotyrosine), and mtDNA damage—particularly vulnerable due to lack of histones and limited repair mechanisms. Elevated 8-hydroxy-2′-deoxyguanosine (8-OhdG) levels further impair respiratory chain function, creating a vicious ROS–mitochondria cycle [47]. The severity of oxidative stress depends on the phase of illness: ROS levels and damage markers increase during depression and mania, while antioxidant enzyme activity may decline in remission. This pattern suggests a mixed state- and trait-dependent nature of markers and is linked to rapid cycling, treatment resistance, and elevated suicide risk [48]. Chronic mitochondrial dysfunction and oxidative stress lead to neurodegenerative processes. ATP deficit impairs axonal transport, synaptic vesicle turnover, and neurotransmitter release, resulting in dendritic atrophy, synapse loss, and neuronal death. MRS studies have shown reduced N-acetylaspartate (NAA)—a marker of neuronal integrity and mitochondrial metabolism—in the prefrontal cortex and hippocampus, while Diffusion Tensor Imaging (DTI) reveals white matter microstructural abnormalities consistent with axonal injury and ROS-dependent demyelination. Activation of Poly(ADP-ribose) Polymerase (PARP) after DNA damage further depletes NAD^+^/ATP resources, exacerbating the energy crisis [49]. The neuroprogression concept posits that accumulation of oxidative damage with relapses shortens remission periods, intensifies cognitive deficits, and reduces treatment effectiveness [50]. Oxidative stress biomarkers (MDA, 8-OHdG, decreased Glutathione (reduced form)-GSH)) correlate with symptom severity and social functioning; elevated levels predict poorer response to lithium and valproate, underscoring the potential of mitochondrial biomarkers in personalized treatment. Interventions targeting mitochondria and redox balance show promising results: lithium and valproate enhance mitochondrial enzyme activity, stabilize membranes, and induce antioxidant enzyme expression; N-acetylcysteine (a GSH precursor) alleviates depressive symptoms; omega-3, coenzyme Q10, and creatine are also under investigation. Lifestyle interventions are significant as well: physical activity activates mitochondrial biogenesis (Peroxisome Proliferator-Activated Receptor Gamma Coactivator 1-alpha-PGC-1α pathway), and diets rich in antioxidants support defense systems [51]. Looking ahead, pharmacogenomic data suggest that variants in genes encoding mitochondrial proteins and antioxidant enzymes modulate treatment response, justifying the integration of molecular information with clinical evaluation and neuroimaging. Multi-omics approaches (genomics, proteomics, metabolomics) should better capture BD heterogeneity and identify predictive biomarkers for precision medicine.

Mitochondrial dysfunction and oxidative stress observed in bipolar disorder should be interpreted within a broader transdiagnostic framework, as similar abnormalities have been consistently reported in other psychiatric conditions, including major depressive disorder and schizophrenia [52]. Altered ATP production, impaired respiratory chain activity, excessive reactive oxygen species generation, and mitochondrial DNA damage represent shared biological mechanisms underlying reduced neuronal resilience and synaptic dysfunction across diagnostic categories. However, in bipolar disorder these alterations appear to be distinguished not by their presence per se, but by their pronounced phase-dependent dynamics and their close interaction with calcium signaling, dopaminergic neurotransmission, and neuroinflammatory pathways [53]. In contrast to major depressive disorder, where mitochondrial abnormalities are often relatively stable, bipolar disorder is characterized by dynamic fluctuations in bioenergetic state across manic, depressive, and euthymic phases, which may contribute to episodic instability and mood switching [54,55]. Moreover, mitochondrial dysfunction in bipolar disorder shows a strong association with neuroprogressive processes, including cumulative oxidative damage, kindling phenomena, and increasing treatment resistance over time. Collectively, these observations support the view that mitochondrial alterations constitute a convergent pathophysiological substrate across psychiatric disorders, whereas their disorder-specific clinical expression in bipolar disorder emerges from interactions with phase-dependent network dysregulation and immune activation rather than from disease-specific mitochondrial defects [56,57].

Recent advances in proteomic and metabolomic profiling suggest that these approaches may contribute to the differentiation of bipolar disorder from other psychiatric conditions, particularly major depressive disorder; however, current evidence indicates that such differentiation relies on multivariate molecular patterns rather than on single disease-specific biomarkers [58]. Both disorders share alterations in inflammatory proteins, oxidative stress markers, and pathways related to energy metabolism, indicating substantial overlap in their molecular architecture. Nonetheless, studies employing untargeted metabolomics have reported differences in lipid metabolism, acylcarnitine profiles, tricarboxylic acid cycle intermediates, and tryptophan–kynurenine pathway metabolites, which appear to be more dynamically regulated and phase-dependent in bipolar disorder than in major depressive disorder [59,60]. Proteomic analyses further suggest that distinct configurations of acute-phase proteins, complement system components, and apolipoproteins may characterize bipolar disorder, particularly when integrated with clinical variables and illness course. Importantly, classification performance improves markedly when proteomic and metabolomic data are combined with clinical features and analyzed using multivariate or machine-learning approaches, supporting a dimensional rather than categorical model of disease biology. Taken together, current findings indicate that proteomics and metabolomics do not yet provide definitive diagnostic markers distinguishing bipolar disorder from major depressive disorder, but they hold significant promise for biological stratification, identification of disease subtypes, and prediction of illness trajectory within a precision psychiatry framework [61,62].

In summary, mitochondrial dysfunction and oxidative stress form the biological foundation of bipolar disorder mechanisms. Energy deficits and oxidative damage impair neuroplasticity, accelerate neurodegeneration, and worsen clinical outcomes. Integrating mitochondrial biomarkers with clinical and neuroimaging assessments may facilitate the development of personalized therapeutic strategies guided by individual biological profiles.

## 4. Genetic and Epigenetic Architecture of Bipolar Disorder

Genetic and epigenetic underpinnings of BD are also gaining prominence, providing critical insight into the molecular mechanisms underlying disease susceptibility and clinical heterogeneity. Genome-Wide Association Studies (GWASs) have revealed numerous risk loci related to neurotransmission, synaptic plasticity, and mitochondrial function, while epigenetic studies indicate that DNA methylation, histone modifications, and non-coding ribonucleic acids (RNAs) dynamically modulate gene expression in response to environmental factors, partly explaining phenotypic heterogeneity [63].

Bipolar disorder is a complex neuropsychiatric condition involving genetic, epigenetic, and environmental factors in its pathogenesis. Early single-gene studies have been supplanted by genome-wide association studies and international consortia research, which have conclusively confirmed the polygenic nature of the disorder. Risk is distributed across thousands of variants of small individual effect, complemented by rarer mutations of stronger impact. Heritability of BD is estimated at 60–80% (family and twin studies). However, genetics alone does not explain phenotypic variability, clinical course, or treatment response. Epigenetic mechanisms—DNA methylation, histone modifications, and regulation by non-coding RNAs—play a crucial role by modulating gene expression depending on biological and environmental context [64].

Over the past decade, GWASs have identified more than 60 loci associated with BD. Many involve genes linked to neuronal signaling and plasticity, including Calcium Voltage-Gated Channel Subunit Alpha1 C (CACNA1C), Ankyrin 3 (ANK3), Odd Oz/Ten-M Homolog 4 (Teneurin-4)-ODZ4, Spectrin Repeat Containing Nuclear Envelope Protein 1 (SYNE1), Glutamate Ionotropic Receptor NMDA Type Subunit 2A (GRIN2A). These variants cluster in pathways regulating calcium channel function, glutamatergic neurotransmission, and synaptic vesicle dynamics, pointing to imbalances in excitatory–inhibitory signaling as a potential mechanism. Other loci involve immune regulation, particularly in the Major Histocompatibility Complex (MHC) region. These data highlight the involvement of both ion channel dysfunction and neuroimmune interactions. Comparative analyses indicate partial overlap in genetic architecture of BD, schizophrenia, and depression, supporting a dimensional approach to psychiatric classification [65].

Polygenic risk scores (PRS), derived from GWAS data, allow for the estimation of cumulative genetic liability. Although they explain only 4–6% of phenotypic variance, PRS correlate with earlier onset, faster cycling, psychotic symptoms, and higher familial burden. Interestingly, PRS also correlate with adaptive traits such as creativity, risk-taking, and educational achievement, suggesting a pleiotropic nature of variants. Rare mutations identified through exome sequencing and Copy Number Variants (CNVs), e.g., in Neurexin 1 (NRXN1) and Contactin-Associated Protein-Like 2 (CNTNAP2), also contribute, implicating genes involved in synapse development and neural network organization [66].

Epigenetic mechanisms demonstrate how genetic liability translates into phenotypes. Epigenetic changes are plastic and influenced by age, stress, infection, or treatment. DNA methylation studies in BD patients have revealed alterations in genes linked to synaptic plasticity, neurogenesis, and circadian rhythms. Examples include changes in Circadian Locomotor Output Cycles Kaput (CLOCK) and Aryl Hydrocarbon Receptor Nuclear Translocator-Like Protein 1 (ARNTL) genes, associated with circadian dysregulation. Some changes are state-dependent, while others are stable and may serve as biomarkers [67].

Mood stabilizers also affect the epigenome. Lithium alters methylation patterns in neurotrophic genes (e.g., BDNF), potentially underlying its neuroprotective and anti-suicidal effects. Valproate acts as a histone deacetylase (HDAC) inhibitor, directly affecting chromatin structure and the expression of numerous mood-regulating genes. Altered histone acetylation and methylation have also been observed in the prefrontal cortex and hippocampus, involving genes related to synaptic transmission and stress response. HDAC inhibitors have shown mood-stabilizing effects in preclinical models, suggesting translational potential for epigenetic research. Non-coding RNAs play an essential role. Dysregulation of microRNAs (e.g., miR-34a, miR-132) affects genes involved in neuronal signaling, stress response, and mitochondrial function. Circulating microRNAs may serve as non-invasive diagnostic and prognostic biomarkers. Long non-coding RNAs (lncRNAs) are also increasingly recognized for modulating transcriptional networks and chromatin structure [68].

The development of multi-omics technologies enables the integration of genomics, epigenomics, transcriptomics, proteomics, and metabolomics, providing a comprehensive view of BD pathophysiology. Transcriptomic analyses of brain tissue reveal consistent disruptions in synaptic, immune, and mitochondrial pathways. Single-cell studies show that risk loci are particularly active in excitatory and inhibitory neurons, as well as in astrocytes and microglia. Risk variants often lie in non-coding regulatory regions (promoters, enhancers), with effects that are cell-type specific. For example, variants near Calcium Voltage-Gated Channel Subunit Alpha1 C (CACNA1C) may disrupt enhancer–promoter interactions and deregulate calcium signaling. Similarly, immune-related variants affect microglial function and inflammatory responses. Proteomic and metabolomic data further confirm the involvement of oxidative stress, neurotransmission dysfunction, and energy metabolism abnormalities [69].

Integration of these findings suggests that polygenic burden shapes molecular networks, which are modulated by epigenetics and manifest as cell- and circuit-specific dysfunctions. Clinically, this implies potential use of PRS and epigenetic signatures as diagnostic and prognostic biomarkers, identification of new therapeutic targets (epigenetic enzymes, RNA regulators), and the advancement of precision psychiatry based on biological stratification of patients [70].

To sum up, the genetic–epigenetic architecture of BD includes multiple small-effect variants and rare high-impact mutations, dynamically modulated by epigenetic processes. Multi-omics research allows mapping of these signals to specific cell types and neural networks, linking genotype to clinical phenotype. Integration of genomics, epigenomics, and systems biology holds the potential to transform BD from a clinically defined condition into a biologically grounded model of circuit dysfunction, paving the way for personalized diagnostics and therapy.

## 5. Neuroimaging Correlates: Linking Molecular Pathways to Brain Circuitry

Increasing neurobiological evidence indicates that in bipolar disorder (BD), immunological disturbances, oxidative stress, mitochondrial dysfunction, and epigenetic mechanisms play key roles, leading to changes in brain structure and function. Neuroimaging techniques allow the capture of processes characteristic of BD directly in the patient’s brain [71]. They enable not only the assessment of structural and network changes but also the analysis of molecular correlates of inflammation and bioenergetic disturbances. The most commonly used methods are MRI, fMRI, PET, and TCD ultrasonography [72]. Increasingly important are multimodal approaches that integrate imaging with molecular biomarkers. MRI studies in BD patients show reproducible, though subtle, changes in brain regions responsible for emotion, cognitive functions, and stress responses. Most frequently, reduced hippocampal volume is observed, particularly in areas related to neurogenesis and stress regulation [73,74]. This phenomenon worsens with illness duration, although lithium may exert a protective effect, slowing atrophy. Similar changes occur in the prefrontal cortex, especially its dorsolateral parts, associated with cognitive deficits and impaired emotional control. Changes also affect the amygdala, which shows both volumetric and connectivity alterations with the prefrontal cortex, hindering emotional regulation. ENIGMA consortium studies reveal widespread, though subtle, structural brain changes in BD, such as reduced cortical thickness in the cingulate gyrus, medial prefrontal cortex, and temporal gyri [75]. These are likely the result of chronic inflammation and mitochondrial dysfunction leading to gradual loss of neuronal integrity. fMRI studies complement these observations, indicating network dysfunction. The most affected networks include the DMN, salience network, and fronto-limbic circuits. In depression, DMN shows hyper-synchronization, favoring rumination, whereas in mania, synchronization is reduced, promoting impulsivity and emotional lability [76,77]. Dysfunction in the salience network impairs switching between DMN and executive networks, leading to abnormal processing of emotional and stress-related stimuli. In fronto-limbic circuits, hyperactivity of the amygdala and reduced prefrontal cortical activity are observed, corresponding to diminished control over emotional responses. These changes have been linked to elevated inflammatory markers (IL-6, TNF-α), confirming the connection between neuroinflammatory processes and altered brain activity [78,79].

Positron Emission Tomography imaging allows for the investigation of molecular processes in the brain. In BD patients, increased microglial activity has been observed, indicative of inflammation and associated with depressive symptom severity and cognitive deficits [80]. FDG-PET shows reduced glucose metabolism in the prefrontal cortex, hippocampus, and amygdala, reflecting bioenergetic disturbances and mitochondrial dysfunction. Modern PET ligands also allow assessment of serotonergic and dopaminergic system function, revealing mechanisms of mood and motivation dysregulation [81]. Importantly, genetic studies show that individuals carrying risk variants in the CACNA1C gene exhibit altered glucose metabolism in fronto-limbic regions, linking genetic burden with neuronal dysfunction. Transcranial Doppler ultrasound complements these methods by evaluating blood flow in major cerebral arteries. While less precise than MRI or PET, TCD ultrasonography is inexpensive, accessible, and allows frequent monitoring [82]. During depression, middle cerebral artery flow velocity decreases, reflecting impaired vascular autoregulation associated with inflammation, oxidative stress, and endothelial dysfunction. In mania, blood flow increases, corresponding to heightened neuronal activity and metabolism. Combining TCD ultrasonography with MRI yields particularly valuable results: MRI captures structural and network activity, while TCD ultrasonography provides dynamic blood flow data [83]. This approach allows linking reduced flow to hippocampal atrophy and decreased prefrontal activity in depression, or increased flow to hyperactivity in fronto-limbic circuits during mania [84,85,86]. Such integration enables testing hypotheses about BD pathophysiology, connecting vascular changes with oxidative stress markers, mitochondrial dysfunction, and structural brain alterations. Integrating imaging and molecular data allows construction of coherent pathophysiological models of BD [87]. Elevated proinflammatory cytokines (IL-6, TNF-α, IL-1β), oxidative stress markers (MDA, 8-OHdG), and mitochondrial indicators (ATP, mtDNA copy number) correlate with hippocampal atrophy, cortical thinning, reduced glucose metabolism, and network connectivity disruptions. Multi-omics data (genomics, epigenomics, proteomics, metabolomics) combined with MRI, PET, and TCD ultrasonography results indicate that genetic burden and epigenetic regulation lead to mitochondrial disturbances and chronic inflammation, which in turn disrupt neural circuits controlling emotion and cognition [88].

From a clinical perspective, this approach opens the door to precision psychiatry. Integrating neuroimaging and biomarkers allows identification of biological patient subtypes, prediction of disease course, and selection of personalized interventions. TCD ultrasonography can serve as a screening and monitoring tool, MRI and PET provide detailed structural and molecular characterization, and their combination allows dynamic patient monitoring and early detection of relapse indicators [89].

In summary, neuroimaging studies in BD reveal a comprehensive view of pathophysiology: from subtle structural changes and network connectivity disruptions, through microglial activation and bioenergetic deficits, to cerebral blood flow abnormalities. Combining imaging and molecular data shows that immunological and mitochondrial disturbances underpin neural network reorganization, serving as key targets for the development of modern, personalized therapies [90].

The associations between molecular biomarkers and neuroimaging findings are presented in Table 1.

This review has several limitations. First, most studies discussed are cross-sectional, which limits causal interpretation and does not allow clear separation of trait-related biological changes from state-dependent alterations occurring during manic or depressive episodes. Longitudinal data across different phases of bipolar disorder are still limited.

Second, bipolar disorder is a clinically heterogeneous condition. Differences in subtype, illness duration, comorbidities, medication exposure, and lifestyle factors may influence molecular biomarkers and neuroimaging findings. In addition, commonly used treatments affect inflammatory pathways, mitochondrial function, and brain structure, which complicates interpretation of disease-specific mechanisms.

Third, neuroimaging studies vary in methodology, including acquisition protocols and data analysis, reducing comparability between studies. Peripheral biomarkers are also influenced by multiple confounding factors and may not fully reflect central nervous system processes.

Moreover, although neuroimaging research in bipolar disorder has expanded substantially, the coverage of MRI and fMRI findings remains uneven. In particular, key areas of the literature addressing white matter integrity, including diffusion-based imaging (DWI/DTI) studies and investigations of myelin abnormalities, are insufficiently integrated [91].

From an integrative perspective, alterations in white matter organization may represent a key convergent pathway through which immune dysregulation and bioenergetic impairment translate into large-scale network dysfunction and clinical manifestations of bipolar disorder [92].

Furthermore, relatively few studies have systematically examined neurobiological and neuroimaging features in offspring of individuals with bipolar disorder [93,94]. Evidence from high-risk populations suggests that subtle alterations in brain structure, functional connectivity, and white matter microstructure may be present prior to the onset of clinical symptoms, indicating potential markers of vulnerability rather than consequences of illness progression or treatment. However, findings remain heterogeneous, highlighting the need for larger, longitudinal studies to clarify their specificity and predictive value [93,95,96].

The multifactorial pathophysiology of bipolar disorder is summarized in Table 2, highlighting key genetic, epigenetic, immunological, mitochondrial, and neuroimaging mechanisms.

Finally, although multimodal and integrative approaches are promising, standardized analytical frameworks and large replicated datasets are still lacking, limiting immediate clinical application.

## 6. Conclusions and Future Directions

Bipolar disorder arises from the interaction of genetic, epigenetic, immune, mitochondrial, and large-scale brain network alterations. Its clinical heterogeneity cannot be explained by a single mechanism; rather, it reflects the dynamic integration of molecular and systems-level processes that evolve across different phases of the illness.

Importantly, many biological alterations observed in bipolar disorder—such as chronic inflammation, oxidative stress, and mitochondrial dysfunction—are not disease-specific, but are also reported in other psychiatric conditions, including major depressive disorder. Current evidence therefore suggests that bipolar disorder is distinguished less by the presence of unique biomarkers and more by the phase-dependent dynamics and network-level integration of shared biological pathways. Consequently, molecular differentiation between bipolar and unipolar depression, particularly during depressive episodes, remains a major unresolved challenge.

Against this background, future research should prioritize longitudinal study designs capable of capturing transitions between manic, depressive, and euthymic states. Equally important is the large-scale validation of candidate biomarkers across genetic, epigenetic, inflammatory, mitochondrial, proteomic, and metabolomic domains. In parallel, further development of multimodal approaches integrating omics data with neuroimaging—supported by advanced multivariate and machine-learning methods—will be essential for identifying biologically meaningful subtypes of bipolar disorder.

From a clinical perspective, although these approaches are not yet ready for routine implementation, integrated molecular and neuroimaging markers hold considerable promise for precision psychiatry. Their future application may improve diagnostic accuracy, reduce misclassification with unipolar depression, enhance patient stratification, and support the development of personalized treatment strategies targeting core pathophysiological mechanisms rather than symptoms alone.

Future research directions and potential clinical applications are summarized in Table 3.

## Figures and Tables

**Table 1:** Associations between Molecular Biomarkers and Neuroimaging Findings in Bipolar Disorder.

Molecular Biomarker/Biological Process	Direction of Change in BD	Neuroimaging Findings	Involved Brain Structures/Networks	Clinical Relevance	References
IL-6, TNF-α, IL-1β	↑ increased	Reduced gray matter volume, cortical thinning	Hippocampus, prefrontal cortex, cingulate gyrus	Symptom severity, poor treatment response, neuroprogression	[11,20,21,25,28,40,56]
IL-18 (NLRP3 inflammasome activation)	↑ increased	Enhanced microglial activity (TSPO PET)	Prefrontal cortex, anterior cingulate cortex, hippocampus	Higher episode frequency, disease progression	[27,28,29,30,41]
MCP-1, CCL2, CCL5, Interleukin-8 (IL-8; CXCL8)	↑ increased	Disrupted functional network integrity	Salience network, default mode network	Emotional dysregulation, mood lability	[11,21,25,28,32]
CX3CL1 (fractalkine)	↓/dysregulated	Impaired neuron–microglia communication	Fronto-limbic circuits	Synaptic plasticity disturbances	[25,31]
Microglial activation	↑ increased	Elevated TSPO ligand binding (PET)	Prefrontal cortex, hippocampus, ACC	Cognitive deficits, depressive symptomatology	[11,15,22,25]
BDNF	↓ reduced	Decreased hippocampal volume	Hippocampus, prefrontal cortex	Cognitive impairment, poor functional recovery	[2,12,51]
IDO activation/kynurenine pathway (quinolinic acid)	↑ increased	Glutamatergic transmission abnormalities	Prefrontal cortex, limbic circuits	Anhedonia, depression, neurotoxicity	[6,20,25,42]
Mitochondrial ATP production	↓ reduced	Decreased glucose metabolism (FDG-PET)	Prefrontal cortex, hippocampus, amygdala	Fatigue, cognitive dysfunction	[23,36,48,49,52]
mtDNA copy number, respiratory chain complexes I and IV	↓ reduced	Lower N-acetylaspartate levels (MRS)	Prefrontal cortex, hippocampus	Neurodegeneration, treatment resistance	[23,36,48,51,52]
Reactive oxygen species, MDA, 8-OHdG	↑ increased	White matter microstructural damage (DTI)	Fronto-limbic pathways	Neuroprogression, rapid cycling	[31,36,47,48,49,52]
Antioxidant systems (GSH, SOD, catalase)	↓ reduced	Axonal integrity disruption	White matter tracts	Impaired social and cognitive functioning	[12,31,47,48,52,57]
CACNA1C genetic risk variants	Risk alleles present	Altered cerebral glucose metabolism	Fronto-limbic circuits	Predisposition to severe disease course	[43,47,59]
Epigenetic alterations (BDNF, CLOCK genes)	Dysregulated	Abnormal DMN rhythmicity	Default mode network, salience network	Sleep disturbances, relapse vulnerability	[2,16,17,44,45]
Cerebral blood flow (TCD ultrasonography measures)	↓ in depression/↑ in mania	Altered middle cerebral artery flow velocity	Global cerebrovascular networks	Phase-dependent biomarker	[14]

Legends: Associations between biomarkers and imaging changes are shown. This table links molecular biomarkers (inflammatory cytokines, oxidative stress markers, mitochondrial dysfunction, and polygenic scores) with structural and functional brain changes detected by MRI, fMRI, PET, and TCD. Abbreviations: 8-OHdG—8-Hydroxy-2′-deoxyguanosine, ATP—Adenosine Triphosphate, FDG-PET—Fluorodeoxyglucose Positron Emission Tomography, fMRI—Functional Magnetic Resonance Imaging, IL-1β—Interleukin 1 Beta, IL-6—Interleukin 6, IL-10—Interleukin 10, MDA—Malondialdehyde, MRI—Magnetic Resonance Imaging, mtDNA—Mitochondrial Deoxyribonucleic Acid, PET—Positron Emission Tomography, PFC—Prefrontal Cortex, PRS—Polygenic Risk Score, TNF-α—Tumor Necrosis Factor Alpha, TSPO—Translocator Protein (18 kDa).

**Table 2:** Risk factors and pathophysiological mechanisms involved in bipolar disorder.

Pathophysiological Mechanism	Key Molecular and Cellular Processes	Neurobiological and Clinical Consequences	References
Genetic and epigenetic susceptibility	Polygenic risk variants (CACNA1C, ANK3, ODZ4); epigenetic regulation of ion channel expression	Increased vulnerability to affective instability	[44,53,54,63,66,79,91]
Neurotransmission and synaptic dysfunction	Dopaminergic hyperactivity; serotonergic and noradrenergic deficits; glutamatergic excitotoxicity (AMPA/NMDA imbalance)	Psychotic symptoms; cognitive and executive dysfunction	[83,84]
Mitochondrial dysfunction and impaired bioenergetics	Disrupted oxidative phosphorylation; ATP depletion; reduced mitochondrial DNA copy number	Neuroprogression; reduced neuronal resilience	[43,50,85]
Oxidative and nitrosative stress	Excessive ROS/RNS production; lipid peroxidation; impaired HDL redox balance; altered fatty acid metabolism	Synaptic and neuronal damage; accelerated brain aging	[29,37,40,41]
Neuroinflammation and microglial activation	Elevated IL-6, TNF-α, and IL-1β levels; chronic microglial priming; cytokine-dependent synaptic alterations	Increased severity of affective episodes; cognitive deficits	[21,22,24,40,78,79]
NLRP3 inflammasome activation	DAMP-induced NLRP3 complex assembly; caspase-1 activation; maturation of IL-1β and IL-18	Chronic neuroinflammation; synaptic dysfunction; mood destabilization	[9,10,11,12,25,28,29,30,41]
Endoplasmic reticulum stress and UPR dysregulation	Activation of PERK, IRE1, and ATF6 pathways; impaired proteostasis; crosstalk with inflammatory signaling	Neuronal vulnerability; impaired emotional regulation; neurodegenerative progression	[20,23,29]
Impaired autophagy	Dysregulated autophagic flux; defective mitophagy; accumulation of damaged proteins and organelles	Reduced neuronal survival; enhanced oxidative stress	[29,37,40]
Immunometabolic alterations	Dysregulation of the kynurenine–tryptophan pathway	Symptom exacerbation; metabolic comorbidities	[33,47]
HPA axis dysregulation	Gene–stress interactions	Episode triggering	[20,34,42]
Circadian rhythm and clock gene disturbances	Sleep deprivation–induced microglial activation; biological rhythm instability	Manic switching; increased relapse risk	[45,52]
Impaired neuroplasticity and neurotrophic signaling	BDNF promoter methylation; disrupted synaptic remodeling	Cognitive deficits	[2,12,46]
Neuroprogression and kindling mechanisms	Recurrent episodes leading to progressive synaptic and structural changes	Increased episode frequency; treatment resistance	[23,34,40]
Structural brain alterations	Volumetric abnormalities of subcortical structures and the hippocampus	Cognitive and emotional dysfunction	[51,52,53,66]
Environmental stress	Interaction with polygenic risk and epigenetic programming	Early disease onset	[20,42,48]
Neuroimaging correlates	TSPO PET imaging of microglial activation	Translational biomarkers	[72,89,90,93,95,96]

Overview of the major risk factors and pathophysiological mechanisms involved in bipolar disorder. List of Abbreviations: ATP—adenosine triphosphate; AMPA—α-amino-3-hydroxy-5-methyl-4-isoxazolepropionic acid receptor; NMDA—*N*-methyl-D-aspartate receptor; BDNF—brain-derived neurotrophic factor; CACNA1C—calcium voltage-gated channel subunit alpha1 C; ANK3—ankyrin G; ODZ4 (TENM4)—teneurin transmembrane protein 4; ROS—reactive oxygen species; RNS—reactive nitrogen species; HDL—high-density lipoprotein; IL-1β, IL-6, IL-18—interleukin-1 beta, interleukin-6, interleukin-18; TNF-α—tumor necrosis factor alpha; NLRP3—NOD-, LRR- and pyrin domain-containing protein 3; UPR—unfolded protein response; PERK—protein kinase RNA-like endoplasmic reticulum kinase; IRE1—inositol-requiring enzyme 1; ATF6—activating transcription factor 6; HPA axis—hypothalamic–pituitary–adrenal axis; TSPO—translocator protein 18 kDa; PET—positron emission tomography.

**Table 3:** Future directions and clinical implications in Bipolar Disorder.

Area	Main Focus	Clinical Relevance	References
Genetic risk	Polygenic risk scores and rare variants	Identification of high-risk individuals and patient stratification	[17,42,43,44,48]
Epigenetic mechanisms	DNA methylation, histone modifications, non-coding RNAs	Prognostic and predictive biomarkers; monitoring treatment effects	[2,16,17,44,45,54,65]
Immune dysregulation	Pro-inflammatory cytokines, microglial activation, NLRP3	Development of immunomodulatory and anti-inflammatory therapies	[11,20,21,25,28,56]
Mitochondrial dysfunction	ATP synthesis, mtDNA integrity, oxidative stress	Novel treatments targeting bioenergetic deficits and neuroprogression	[23,36,46,48,51,52]
Neuroimaging biomarkers	MRI, fMRI, PET, TCD ultrasonography	Objective markers of disease stage, relapse risk, and treatment response	[14,15,71,75,81,88]
Multimodal approaches	Integration of imaging with molecular biomarkers	Improved disease characterization and monitoring	[21,28,71,73,93]
Multi-omics strategies	Genomics, epigenomics, proteomics, metabolomics	Identification of biological subtypes of bipolar disorder	[24,42,64,67,68]
Predictive models	Biomarker-based algorithms	Prediction of relapse and treatment outcome	[50,61,63,69,87]
Personalized psychiatry	Biological patient stratification	Individualized treatment strategies	[58,59,60,63,71]
Clinical translation	Implementation of biomarker-guided care	Earlier intervention and improved long-term outcomes	[21,58,59,63,71]

Table 3 presents the main future research directions and clinical implications in bipolar disorder. List of abbreviations: ATP—Adenosine Triphosphate, DNA—Deoxyribonucleic Acid, fMRI—Functional Magnetic Resonance Imaging, MRI—Magnetic Resonance Imaging, mtDNA—Mitochondrial DNA, NLRP3—NLR Family Pyrin Domain Containing 3, PET—Positron Emission Tomography, RNA—Ribonucleic Acid, TCD—Transcranial Doppler Ultrasonography.

## Data Availability

All data and analysis are available within the manuscript or upon submitting a request to the corresponding author.

## Abbreviations

The following abbreviations are used in this manuscript:

8-OHdG	8-Hydroxy-2′-deoxyguanosine
ACC	Anterior Cingulate Cortex
AI	Artificial Intelligence
AMPA	α-Amino-3-hydroxy-5-methyl-4-isoxazolepropionic acid receptor
ANK3	Ankyrin G
ARNTL	Aryl Hydrocarbon Receptor Nuclear Translocator-Like Protein 1
ATP	Adenosine Triphosphate
BD	Bipolar Disorder
BDNF	Brain-Derived Neurotrophic Factor
CACNA1C	Calcium Voltage-Gated Channel Subunit Alpha1 C
CCL2	C-C Motif Chemokine Ligand 2
CCL5	C-C Motif Chemokine Ligand 5
CLOCK	Circadian Locomotor Output Cycles Kaput
CNTNAP2	Contactin-Associated Protein-Like 2
CNV	Copy Number Variant
CRP	C-Reactive Protein
CX3CL1	C-X3-C Motif Chemokine Ligand 1 (Fractalkine)
CXCL8	C-X-C Motif Chemokine Ligand 8
DALY	Disability-Adjusted Life Years
DAMPs	Damage-Associated Molecular Patterns
DMN	Default Mode Network
DNA	Deoxyribonucleic Acid
DTI	Diffusion Tensor Imaging
EAAT1/2	Excitatory Amino Acid Transporter 1/2
ENIGMA	Enhancing NeuroImaging Genetics through Meta-Analysis
ER	Endoplasmic Reticulum
FDG-PET	Fluorodeoxyglucose Positron Emission Tomography
fMRI	Functional Magnetic Resonance Imaging
GFAP	Glial Fibrillary Acidic Protein
GSH	Glutathione (reduced form)
GWAS	Genome-Wide Association Study
HDAC	Histone Deacetylase
HDL	High-Density Lipoprotein
HPA axis	Hypothalamic–Pituitary–Adrenal axis
IDO	Indoleamine 2,3-Dioxygenase
IFN-γ	Interferon Gamma
IL-1β	Interleukin 1 Beta
IL-6	Interleukin 6
IL-8	Interleukin 8
IL-10	Interleukin 10
IL-17	Interleukin 17
IL-18	Interleukin 18
IRE1	Inositol-Requiring Enzyme 1
lncRNA	Long Non-Coding RNA
MCP-1	Monocyte Chemoattractant Protein 1
MDA	Malondialdehyde
MHC	Major Histocompatibility Complex
MRI	Magnetic Resonance Imaging
MRS	Magnetic Resonance Spectroscopy
mtDNA	Mitochondrial DNA
NAA	N-Acetylaspartate
NMDA	*N*-Methyl-D-Aspartate receptor
NLRP3	NOD-, LRR- and Pyrin Domain-Containing Protein 3
NRXN1	Neurexin 1
ODZ4 (TENM4)	Odd Oz/Ten-M Homolog 4 (Teneurin Transmembrane Protein 4)
PARP	Poly(ADP-ribose) Polymerase
PET	Positron Emission Tomography
PFC	Prefrontal Cortex
PGC-1α	Peroxisome Proliferator-Activated Receptor Gamma Coactivator 1-alpha
PRS	Polygenic Risk Score
RANTES	Regulated on Activation, Normal T-cell Expressed and Secreted
RNA	Ribonucleic Acid
ROS	Reactive Oxygen Species
RNS	Reactive Nitrogen Species
S100B	S100 Calcium-Binding Protein B
SOD	Superoxide Dismutase
SYNE1	Spectrin Repeat Containing Nuclear Envelope Protein 1
TCD	Transcranial Doppler Ultrasonography
TGF-β	Transforming Growth Factor Beta
TNF-α	Tumor Necrosis Factor Alpha
TSPO	Translocator Protein 18 kDa
UPR	Unfolded Protein Response
WHO	World Health Organization
